# Foraging of *Psilocybe* basidiocarps by the leaf-cutting ant *Acromyrmex lobicornis* in Santa Fé, Argentina

**DOI:** 10.1186/2193-1801-2-254

**Published:** 2013-06-05

**Authors:** Virginia E Masiulionis, Roland WS Weber, Fernando C Pagnocca

**Affiliations:** Instituto de Biociências, UNESP – Univ Estadual Paulista, Campus de Rio Claro, SP. Centro de Estudos de Insetos Sociais, Rio Claro, SP 13506-900 Brazil; Esteburg-Obstbauzentrum Jork, Moorende 53, Jork, 21635 Germany

**Keywords:** *Acromyrmex lobicornis*, Basidiocarps, Coprophilous fungus, *Deconica coprophila*, Forage behaviour, Leaf-cutting ants, *Psilocybe coprophila*

## Abstract

**Background:**

It is generally accepted that material collected by leaf-cutting ants of the genus *Acromyrmex* consists solely of plant matter, which is used in the nest as substrate for a symbiotic fungus providing nutrition to the ants. There is only one previous report of any leaf-cutting ant foraging directly on fungal basidiocarps.

**Findings:**

Basidiocarps of *Psilocybe coprophila* growing on cow dung were actively collected by workers of *Acromyrmex lobicornis* in Santa Fé province, Argentina. During this behaviour the ants displayed typical signals of recognition and continuously recruited other foragers to the task. Basidiocarps of different stages of maturity were being transported into the nest by particular groups of workers, while other workers collected plant material.

**Conclusions:**

The collection of mature basidiocarps with viable spores by leaf-cutting ants in nature adds substance to theories relating to the origin of fungiculture in these highly specialized social insects.

**Electronic supplementary material:**

The online version of this article (doi:10.1186/2193-1801-2-254) contains supplementary material, which is available to authorized users.

## Background

Ants in the genera *Atta* and *Acromyrmex* (Hymenoptera: Formicidae: Attini) are eusocial insects known as leaf-cutting ants because members of their foraging caste (foragers) cut and carry fresh plant material, including leaves, flowers, fruit and seeds, into the nest (Weber 1972). These activities are part of the foraging behaviour which comprises searching, selecting, cutting and transporting of the plant matter (Wilson 1971,1980). Plants to be cut are carefully selected according to physical parameters such as hardness or water content of leaves (Bowers and Porter 1981 Waller 1982 Nichols-Orians and Schultz 1989) as well as chemical characteristics such as toxins, terpenoids or antifungal compounds (Rockwood 1975,1976 Hubbell et al. 1984 Howard 1988). Different attine ant species may show a preference for foraging on monocotyledons, dicotyledons, or both (Fowler et al. 1990 Lopes 2005). In the nest, the freshly cut material is extensively processed (Diniz and Bueno 2009), followed by inoculation with a basidiomycete fungus such as *Leucoagaricus gongylophorus* (A. Möller) Singer. The fungus garden thus established serves as the source of food for the colony and is carefully maintained (Weber 1972 Quinlan and Cherrett 1979). The association between *Leucoagaricus* and leaf-cutting ants is considered to be mutually and obligately symbiotic (Weber 1972).

*Acromyrmex lobicornis* Emery is a leaf-cutting ant species distributed from subtropical areas in southern Brazil and Bolivia (23° S) through northern Patagonia, Argentina (44° S) (Farji-Brener and Ruggiero 1994). This species shows a preference for dicotyledonous plants, monocotyledons being collected only sporadically (Franzel and Farji-Brener 2000). We were therefore surprised to observe foragers of *A. lobicornis* cutting and carrying basidiocarps of a coprophilous fungus.

## Methods

Field observations of *A. lobicornis* collecting fungal basidiocarps were made on 9 January 2010 at 10:35 am in Santurce (Santa Fé province, Argentina; 30°11′16.14″S; 61°10′24.35″W). Photographs and video sequences were taken with a Sony Cyber-Shot DSC-W120 camera. Pure cultures of the fungus were obtained by attaching mature basidiocarps to the lid of a Petri dish with a streak of vaseline jelly, permitting basidiospores to be released onto an agar plate of potato dextrose agar (PDA) augmented with penicillin G and streptomycin sulphate (each at 200 mg l^-1^). After 48 h, samples of growing mycelium were excised from the margins of basidiospore deposits with a fine needle, and transferred to fresh PDA plates. Mycelium of a representative 7-d-old PDA culture was used for DNA extraction, PCR amplification and sequencing of the internal transcribed spacer (ITS) region of ribosomal DNA as described by Weber (2011). Sequence searches were performed in GenBank using the BLASTN function (Zhang et al. 2000).

## Results and discussion

**Figure 1:**
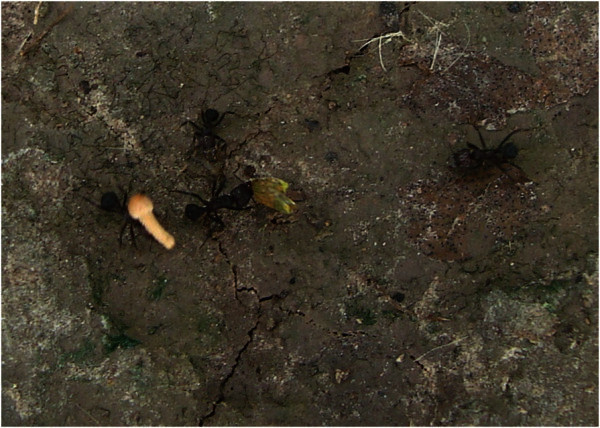
**Worker (forager) of*Acromyrmex lobicornis*carrying an immature basidiocarp of*Psilocybe coprophila*.**

**Figure 2:**
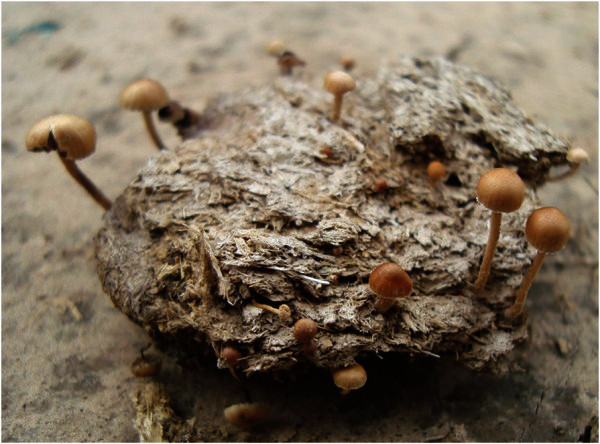
**Basidiocarps of*Psilocybe coprophila*on mature cow dung at the observation site, 9 Jan. 2010.**

Observations of a foraging trail of *A. lobicornis* showed that one group of workers was collecting pieces of dicotyledonous plants whilst another group was cutting and carrying fungal fruit-bodies to the nest (Figure 1). These basidiocarps had grown on the surface of several pats of cow dung (Figure 2) located 50–70 m away from the nest. During a 5-min period, 10 ants entered their nest carrying entire basidiocarps or parts of them. Both immature and fully expanded basidiocarps were collected (see Additional file 1). Further documentation is available from the corresponding author upon request.

**Figure 3:**
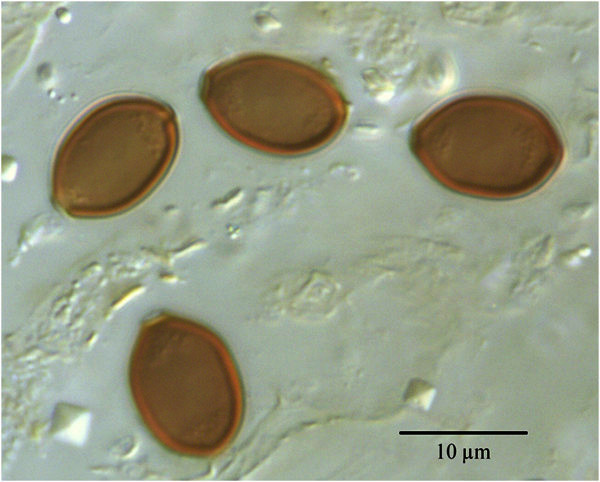
**Basidiospores of*Psilocybe coprophila*from a mature fruit-body collected at the observation site.**

Basidiocarps were 10–30 mm long, and fully expanded caps measured 6–10 mm diam. Basidiospores were produced abundantly by mature basidiocarps. They were thick-walled, brown, flattened, somewhat angular in outline, and possessed a basal scar and an apical germ pore (Figure 3). They measured 11.0-13.0 × 7.8-9.1 × 6.6-8.0 μm. Keys of coprophilous fungi (Watling and Gregory 1987 Richardson and Watling 1997) permitted identification of the coprophilous fungus as *Psilocybe coprophila* (Bull.: Fr.) Kumm. [syn. *Deconica coprophila* (Bull.: Fr.) Karst.]. The ITS rDNA sequence, deposited in GenBank as accession number JX235960, confirmed *P. coprophila* (accession AJ519795) to be the closest available match, showing a sequence identity at 591 out of 595 nt overlap.

Although *A. lobicornis* is known to harvest a variety of plants (Franzel and Farji-Brener 2000), fungi have not been described as being part of its collections before. Indeed, to the best of our knowledge the study by Lechner and Josens (2012) is the only previous observation of any leaf-cutting ant species collecting fungal basidiocarps. In that study, fruit bodies of *Agrocybe cylindracea* (DC) Maire that had grown on the surface of *Populus* bark in Buenos Aires (Argentina) were collected by *Acromyrmex lundii* Guérin-Méneville. Further, Lechner and Josens (2012) were able to demonstrate that *A. lundii* incorporated *A. cylindracea* basidiocarp material into its fungus gardens under laboratory conditions.

It is not known why *Acromyrmex* ants should forage on *P. coprophila* or *A. cylindracea*, given that they cultivate their *Leucoagaricus* diet in their nest. Further, although fungal mycelium is a suitable food source in being rich in carbohydrates and proteins (Mueller et al. 2001), few non-leaf-cutting ants seem to have exploited this. *Euprenolepis procera* Emery from South-East Asian rainforests is the only known ant species specializing in the collection of fungal fruit bodies as the main diet (Witte and Maschwitz 2008).

The nutritional interactions between *Leucoagaricus* and attine ants are largely unknown, although enzymatic contributions by both symbiotic partners to the degradation and processing of the collected plant material are beginning to be revealed (Richard et al. 2005 Silva et al. 2006). In addition, fungus gardens are subject to contamination by microbes originating from soil and plant material (Carreiro et al. 1997 Pagnocca et al. 2012). These may be controlled by grooming behaviour and by antibiotics produced by bacteria (*Pseudonocardia* spp.) colonising the cuticle of attine ants Poulsen and Currie (2010). However, except for the laboratory study by Lechner and Josens (2012) there is no record of the presence of mushroom-type basidiomycetes in fungus gardens other than *Leucoagaricus* itself.

Our observation of basidiocarp collections by attine ants raises obvious questions relating to the origin of fungiculture. The ‘Consumption First’ model (Weber 1972) postulates that a fungus species which was at first collected and directly consumed by ants might have become a mutualistic symbiont over time, once the ants had become capable of cultivating it and transmitting it to their offspring. More detailed field observations should be conducted to assess the frequency of basidiocarp collection by *A. lobicornis* in nature. Fungus gardens of basidiocarp-collecting colonies should be analysed for the presence of these basidiomycetes using microbiological or molecular biological methods.

## Electronic supplementary material

Additional file 1: **Video sequence showing the collection of basidiocarps of*Psilocybe coprophila*by*Acromyrmex lobicornis*ants.** (MP4 17 MB)
